# No-Reference-Based and Noise Level Evaluations of Cinematic Rendering in Bone Computed Tomography

**DOI:** 10.3390/bioengineering11060563

**Published:** 2024-06-02

**Authors:** Jina Shim, Youngjin Lee

**Affiliations:** 1 Department of Diagnostic Radiology, Severance Hospital, 50-1, Yonsei-ro, Seodaemun-gu, Seoul 03722, Republic of Korea; eoeornfl@yuhs.ac; 2 Department of Radiological Science, Gachon University, Incheon 21936, Republic of Korea

**Keywords:** bone computed tomography, 3D computed tomographic image, cinematic rendering, no-reference-based evaluation, noise level evaluation

## Abstract

Cinematic rendering (CR) is a new 3D post-processing technology widely used to produce bone computed tomography (CT) images. This study aimed to evaluate the performance quality of CR in bone CT images using blind quality and noise level evaluations. Bone CT images of the face, shoulder, lumbar spine, and wrist were acquired. Volume rendering (VR), which is widely used in the field of diagnostic medical imaging, was additionally set along with CR. A no-reference-based blind/referenceless image spatial quality evaluator (BRISQUE) and coefficient of variation (COV) were used to evaluate the overall quality of the acquired images. The average BRISQUE values derived from the four areas were 39.87 and 46.44 in CR and VR, respectively. The difference between the two values was approximately 1.16, and the difference between the resulting values increased, particularly in the bone CT image, where metal artifacts were observed. In addition, we confirmed that the COV value improved by 2.20 times on average when using CR compared to VR. This study proved that CR is useful in reconstructing bone CT 3D images and that various applications in the diagnostic medical field will be possible.

## 1. Introduction

Imaging systems using X-rays are essential in the field of diagnostic medical imaging [1,2]. This imaging system uses a detector to acquire information about how X-rays penetrate and attenuate materials (information for linear attenuation coefficient) as a final image [3]. Because images acquired with X-rays in one direction may result in overlapping structures, computed tomography (CT) using 360-degree projection data and 3D reconstruction is widely used [4]. With the advancement of various technologies, excellent anatomical information can be achieved with CT using various image acquisition methods [5,6,7].

Hardware and software advances in computed tomography (CT) have made it possible to obtain volume datasets with isotropic submillimeter voxel sizes [8,9]. This increases the resolution of multiplanar reformation (MPR) images to the extent that they are similar to cross-sectional images, enabling preoperative treatment planning for patients who were involved in traffic accidents, especially as 3D imaging post-processing becomes possible, which helps understand structural morphology [10,11]. Volume rendering (VR) is one of the most common methods for observing CT images in 3D. VR maintains the characteristics of the initially obtained image in the final result because it is processed in voxel units.

Because the CT number of bones is much higher than that of other tissues, 3D imaging techniques, including VR, can be used for visualization. High-quality 3D images can be comprehensively visualized and can help express and interpret anatomical relationships [12,13,14,15,16]. These images enable image-based treatment planning for orthopedic surgery in various situations. This is because 3D images with improved quality can help visualize the anatomical details of various aspects, thus increasing doctors’ understanding of the scope of surgery. In 3D images, a realistic presentation can provide information and understanding that cannot be obtained from cross-sectional or plain imaging. This can help the surgeon understand the anatomical structure, increase the success rate of surgery, and reduce the likelihood of complications by shortening the time of surgery.

Despite these advantages, VR also has several limitations. Because 3D images inevitably require volume understanding through 2D planar images, overlapping of different anatomical structures and pathological factors can affect the diagnosis. This means that there is a limit to expressing the relationships between spaces that can occur by expressing the volume data in a 2D planar image. Despite the advantages of 3D imaging in diagnostic medicine, VR is not used alone but in conjunction with MPR imaging. In addition, high HU is better separated from low-HU-range anatomical structures in VR, which is beneficial for a better understanding of anatomical structures. However, it is difficult to understand the relationship of anatomical structures with high HU but similar HU ranges because they all look similar, which can lead to errors in recognizing anatomical structures due to the lack of representation of depth relationships.

Cinematic rendering (CR) has been developed to overcome the limitations of conventional VR technology. CR is a new 3D imaging technique inspired by the success of the animated movie industry, and its name is based on the creation of very realistic characters. This technique allowed, for the first time, the production of medical VR images with improved image quality and spatial resolution [9,17]. CR can be used by physicians to study the anatomy of patients and explain specific details of the procedure. Pictorial reviews and qualitative evaluations were conducted to determine the usefulness of this technology in clinical practice [18]. In particular, CR-based images can be used to deliver more specific visual information in virtual anatomy classes, and their applicability to bone CT images has been proven [18,19]. Rowe et al. used CR technology to observe the calvarium, maxillofacial structures, and skull base in more detail and demonstrated that the lesion occurred in the skeleton through shadowing of the edge area [19].

Most studies related to CT images using CR technology applied only qualitative evaluation of the reader because it is difficult to use a reference, and there are currently few studies that have evaluated changes in spatial resolution or differences in noise using quantitative evaluation methods. The noise level of medical images is mainly calculated using coefficient of variation (COV) or signal-to-noise ratio evaluation parameters. In addition, the spatial resolution of CT images is mainly evaluated using full width at half maximum values measured using point area or similarity evaluation based on reference images. It is very important to measure both the noise level and spatial resolution simultaneously; Mittal et al. proposed a method for evaluating the overall image quality without a reference image [20]. Among no-reference-based image evaluation methods, the blind/referenceless image spatial quality evaluator (BRISQUE) is representative. The BRISQUE is a state-of-the-art no-reference-based evaluation method that extracts natural scene statistics features from distorted images and uses support vector machines. This evaluation method was modeled based on the relationship between natural image distortion and pixel statistics, and research results regarding the possibility of application to CT images were derived [21]. BRISQUE parameters can also be actively used to evaluate the final results of new CT imaging technology.

**Figure 1:**
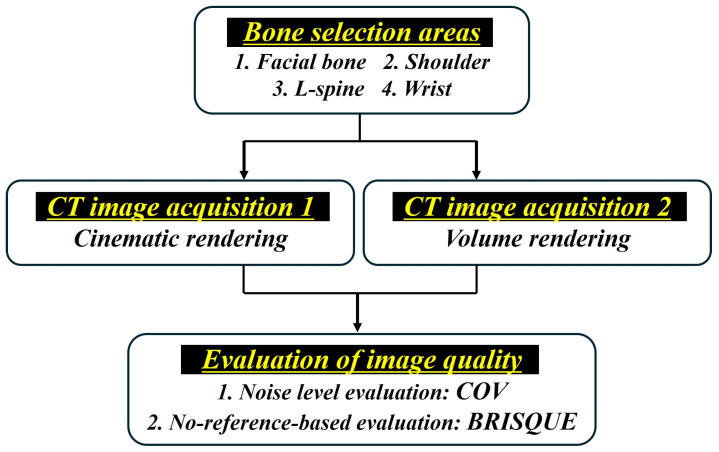
Schematic diagram of the overall flowchart of the study. The flow from the bone selection process in four areas to the evaluation method of image quality after applying cinematic rendering (CR) and volume rendering (VR) techniques is shown.

Thus, the purpose of this study was to quantitatively compare the CT image quality from CR and conventional VR in various bone regions. We acquired bone CT images from four areas (facial bone, shoulder, lumbar spine (L-spine), and wrist) using CR and VR technology and quantitatively evaluated the image quality of all images. As quantitative evaluation factors, we will use COV and BRISQUE to analyze the noise level and overall image quality of CR and VR images. The overall flowchart of this study is shown in Figure 1.

## 2. Materials and Methods

### 2.1. Patient Selection

This study included four consecutive adult patients (3 women, 1 men; mean age ± SD, 69.75 ± 8.84 years) who underwent bone CT examinations (such as facial bone, shoulder, L-spine, and wrist CT scans) in April 2022. An image was selected from a patient whose objective was to examine the bones, as bone CT is an effective method for identifying fractures in bones. Furthermore, metal artifacts are particularly disruptive in three-dimensional imaging, so images of patients whose effects could be observed were selected. All patients underwent CT for the indicated reasons. The clinical indications were internal fixation for comminuted distal radius fracture, posterior decompression, instrumented fusion at the lower lumbar (L_4_–L_5_) region, old nose fracture, and postoperative course after total shoulder arthroplasty.

This study was conducted retrospectively and according to the guidelines of the Declaration of Helsinki and approved by the Institutional Review Board of Severance Hospital (4-2023-0169).

### 2.2. CT scanning Methods

CT scans of the facial bones, shoulder, L-spine, and wrist were performed using Revolution CT (GE Healthcare, Milwaukee, WI, USA), Somatom Force, Somatom Definition Flash (Siemens Healthcare, Forchheim, Germany), and Brilliance iCT (Philips Healthcare, Cleveland, OH, USA). The scanning parameters for each CT scan are listed in Table 1. The scan ranges per CT prescription were included in the entire scan region, and especially for the shoulder, replacement devices were all included in the image. VR and CR reconstructions were performed at a multimodality workstation using the syngo.via v. VB40B program (Siemens Healthineers, Erlangen, Germany). In each case, 3D reconstructions were generated using both VR and CR techniques with identical fields of view, HU ranges, opacity settings, and anteroposterior view. In the case of VR, voxel values below 133 HU are not visualized with an opacity of 0%. Instead, the opacity is incrementally increased to 87% for 133–235 HU, and then to 100% for 235–354 HU. Finally, 100% opacity is displayed in all-white for 354 HU and above. In the case of CR, if the voxel value is less than 62 HU, the opacity is not visualized as 0%. Instead, for 62–99 HU, the opacity is gradually increased to 5%. For 99–127 HU, the opacity is gradually increased to 8%. For 127–195 HU, the opacity is gradually increased to 12%. The opacity is gradually increased to 34% for values between 127 and 195 HU, to 99% for values between 195 and 263 HU, and to 100% for values above 263 HU, in all-white. The mean volumetric CT dose indices for the four types of bone CT examinations (facial bone, shoulder, L-spine, and wrist) were 13.11, 8.76, 18.90, and 6.91 mGy, respectively, while the corresponding dose length products were 352, 227, 752, and 155 mGy*cm, respectively.

### 2.3. Quantitative Evaluation of Image Quality

In this study, the BRISQUE parameter, a no-reference-based evaluation variable proposed by Mittal et al., was used to analyze overall CT image quality [20]. The BRISQUE is calculated based on the no-reference image quality assessment approach and on the hypothesis that it is a statistical characteristic that changes depending on the presence of image distortion. This parameter includes the statistical properties hidden in a natural image using mean subtraction and contrast normalization (MSCN) pre-processing. When MSCN pre-processing is performed on a natural image, the histogram of pixels has a Gaussian distribution. The BRISQUE formula was developed based on the principle of matching a generalized Gaussian distribution (GGD) to the histogram of an MSCN-processed image. $f_{GGD}\left(x;a,\sigma^{2}\right)$, which can represent the range of distorted image statistics using GGD and can be expressed using the gamma function (Δ) as follows [20,22]:

(1) fGGDx;a,σ2=α2βΔ1αexp−xβα

(2) $$\Delta \left(a\right)=\int_{0}^{\infty }t^{a-1}e^{-t}dt\;\left(a>0\right)$$

(3) β=σΔ1αΔ3α

where a indicates the shape parameter that controls the distribution of $\sigma^{2}$.

**Figure 2:**
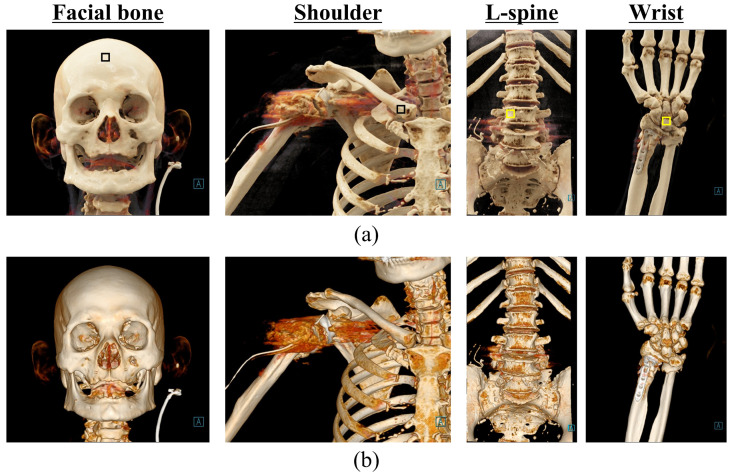
Results obtained using (**a**) cinematic rendering and (**b**) volume rendering techniques. Regions of interest (ROIs) for calculating the coefficient of variation (COV) are indicated (black and yellow rectangular areas).

The COV was used as a factor to evaluate the noise level of images acquired with CT imaging technologies. The region of interest (ROI) for deriving the COV was set for each CT image as a region of uniform shape as much as possible (black and yellow rectangular areas in Figure 2). The formula for calculating the COV is as follows:

(4) $$COV=\frac{\sigma_{T}}{S_{T}}$$

where $S_{T}$ and $\sigma_{T}$ are the mean and standard deviation of the target region, respectively.

For CR and VR data in all bone areas, the average BRISQUE and COV were measured when 10 HU values were adjusted, and the error was calculated using standard deviation.

## 3. Results and Discussion

**Figure 3:**
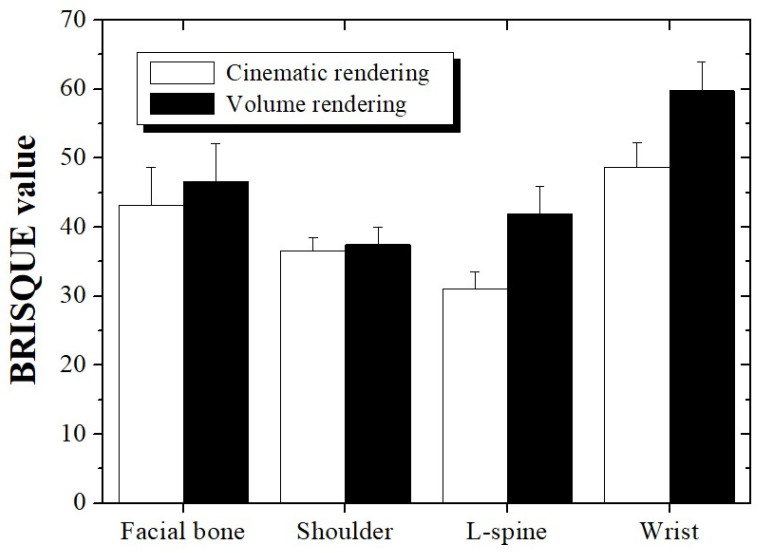
Blind/referenceless image spatial quality evaluator (BRISQUE) results measured using CT images by region, applying cinematic and volume rendering technologies.

Figure 2 shows a bone CT image obtained using CR and VR technologies. The BRISQUE results obtained using Figure 2 are shown graphically in Figure 3. The BRISQUE results obtained using CR were 43.11 ± 5.60, 36.58 ± 1.96, 31.10 ± 2.37, and 48.67 ± 3.55 for the facial bone, shoulder, L-spine, and wrist scans, respectively, and those for the CT images obtained using VR were 46.62 ± 5.54, 37.47 ± 2.59, 41.89 ± 3.98, and 59.78 ± 4.17, respectively. The average BRISQUE values of the four scan datasets were derived as 39.87 and 46.44 when the CT images were reconstructed using CR and VR technologies, respectively. As a result, we were able to confirm that the BRISQUE value improved by approximately 1.16 times compared to VR when CR was applied to CT image reconstruction.

Bone regions in CT images have a higher contrast than soft tissue, which is useful for visualization using 3D imaging post-processing techniques. High-quality 3D images are advantageous for surgeons and patients for understanding and interpreting anatomical relationships. In this context, CR, a new 3D technology, has shown positive clinical responses because of its realistic image representation. Therefore, a study comparing the clinical usefulness of CR and VR and performing image evaluations has been reported. However, because there is no gold standard in the image evaluation of CR and VR, visual evaluation is derived as in most research results [19,23,24,25,26,27]. This study used BRISQUE, a no-reference-based evaluation parameter that can evaluate the overall CT image quality without using a gold-standard image. The BRISQUE parameters have been verified in the field of radiology imaging and can predict the clinical usefulness of CR images and estimate the quality of CR images compared to that of VR images [28]. The results of this study confirmed that CR, which was developed to improve the quality of CT images, had a higher spatial resolution than VR in a no-reference-based evaluation. In addition, this result showed a tendency similar to the results of other studies, in which CR performed better than VR in qualitative evaluations [9,10,17].

The BRISQUE results showed that the CR values were low in all anatomical regions, especially in the L-spine. The L-spine is not significantly affected by noise if the radiation dose is sufficient; therefore, we believe that the use of CR technology with good spatial resolution for CT image reconstruction is appropriate for diagnosis. In addition, metal artifacts were observed in the L-spine and wrist-scanned images. In both cases, we observed that the difference between the CR and VR BRISQUE results increased significantly compared to that in the other areas. The difference between the CR and VR BRISQUE results in the L-spine and wrist area was 25.75 and 18.58%, respectively. Thus, we proved that CR is more useful when scanning the CT images of patients with implanted surgical instruments that can generate metal artifacts.

**Figure 4:**
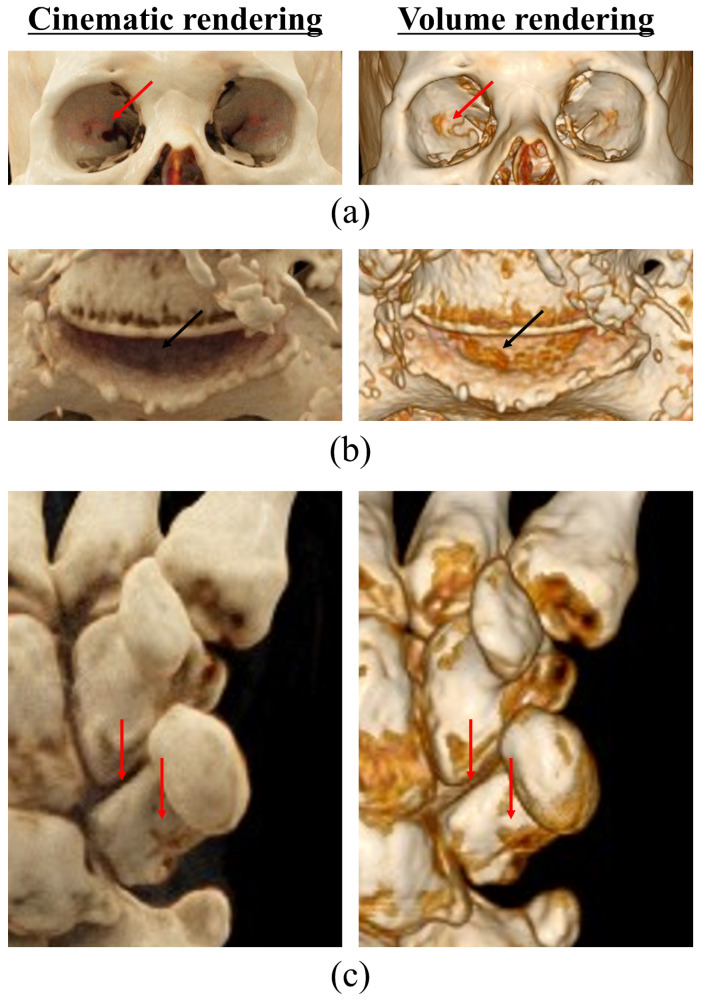
CT images of each enlarged reconstruction technique for comparative observation of depth information: (**a**) facial bone, (**b**) L-spine, and (**c**) wrist scans. The depth information of the part indicated by the red arrows are visually observed better when cinematic rendering technology is applied.

When comparing the CR- and VR-based CT images of the orbital region of the facial bone, the difference in the depth of the image was confirmed. This is because VR images formed their value with one light per pixel, whereas CR images formed one pixel value with billions of lights interacting across voxels [29]. CR images also consider the lighting effects of neighboring voxels and produce reflection and shadow effects (Figure 4). When interpreting 3D images, it is necessary to understand the volume data in flat images. Therefore, reflection and shading effects clarify the relationship between depth information and anatomical structures, enabling accurate spatial relationship evaluation [18,30].

**Figure 5:**
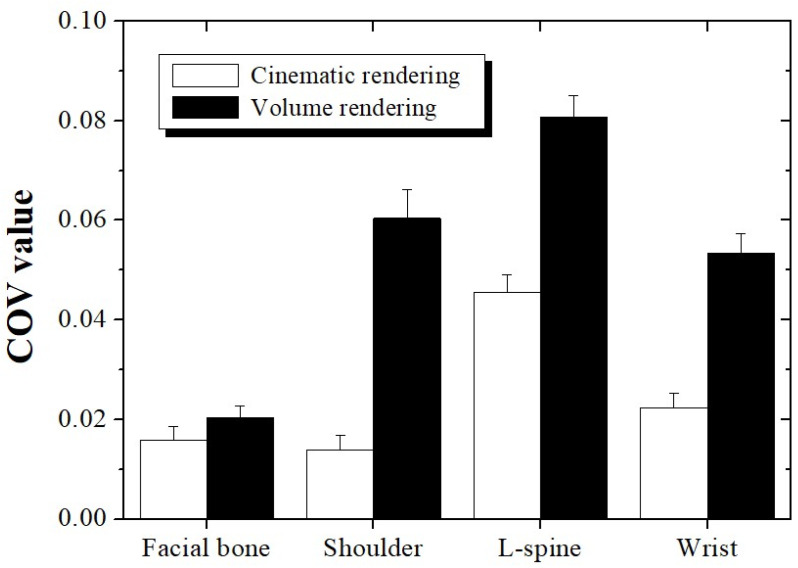
Coefficient of variation (COV) measured using CT images by region, applying cinematic and volume rendering technologies. The regions of interest for the COV measurements are shown in Figure 2.

CR technology has been proven by many studies to improve the quality of CT images compared to conventional 3D methods. In this study, CR and VR technologies were compared using the BRISQUE in terms of overall CT image quality. Additionally, we utilized the COV to quantitatively evaluate the noise. Figure 5 shows a graph of the COV results for each 3D image reconstruction technique using the four types of bone CT images. The COV values for the facial bone, shoulder, L-spine, and wrist CT images obtained using the CR technology were 0.016 ± 0.0027, 0.014 ± 0.0028, 0.046 ± 0.0035, and 0.022 ± 0.0028, respectively, and those using VR technology were 0.020 ± 0.0024, 0.060 ± 0.0058, 0.081 ± 0.0044, and 0.054 ± 0.0038, respectively. The average COV values for the four scan datasets were derived as 0.024 and 0.054 when the CT images were reconstructed using the CR and VR technologies, respectively. As a result, we confirmed that the COV value was improved about 2.20 times compared to VR when CR was applied to CT image reconstruction. The COV is a representative factor that can quantitatively evaluate the noise levels of medical images. We believe that CR technology will be effective for CT images in terms of noise and spatial resolution as the trend of the COV results is similar to that of the BRISQUE.

Unlike VR, CR can be useful in post-processing images where noise occurs due to insufficient tube current. Especially in the emergency room, when a CT scan is performed with a cast on an extremity, a lot of noise is generated in the bone image due to the thick cast. Also, when a fracture in the shoulder area is examined with the hand on the stomach, the image quality is degraded due to the increased effective thickness. This noise and image degradation can make it difficult to distinguish fracture lines and separate bone fragments; therefore, CR, which was found to be effective in terms of noise and spatial resolution in this study, can be useful in such cases.

In this study, we demonstrated that bone CT images using CR technology can simultaneously reduce overall image quality and noise level. When the BRISQUE factor was used in a CT imaging study based on actual experiments where a gold standard could not be established, images based on CR technology showed excellent image quality characteristics. We confirmed that CR technology can significantly reduce the noise level compared to CT images based on VR technology. These advantages of CR technology are expected to greatly contribute to improving depth perception and surface sharpness, which are among the factors commonly evaluated in the 3D imaging field [31]. In addition, it can be expected that the quantitative evaluation results can improve photo-realism when reconstructing 3D CT images using CR technology. Furthermore, we expect that higher-quality results will be obtained when image processing technology that combines various denoising and deblurring algorithms is additionally used as a post-processing method for CR technology images [32].

**Figure 6:**
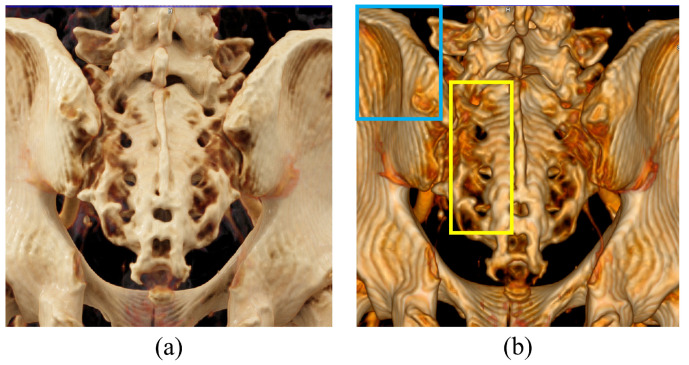
The 3D visualization obtained through a 3 mm coronal CT image: (**a**) cinematic rendering and (**b**) volume rendering. Two distinct rendering techniques were employed to visualize the posterior aspect of the sacrum. In the area designated by the blue box, a pronounced wave-like artifact is observed in the iliac fossa. The yellow box highlights the openings of four sacral foramina, which are challenging to distinguish from the surrounding anatomical structures. The image parameters were set at 100 kV, 240 mAs, with an I30f kernel and an admire level of 2.

Figure 6 shows a VR and CR post-processed image of the back of the sacrum. As shown in Figure 6, the anatomical representations shown are different in CR than in VR. In the case of the sacral foramen, the four foramen pairs are clearly visible in the CR image as dark oval openings amidst the lighter parts of the bone structure. In VR, however, the foramen openings are difficult to distinguish from the surrounding anatomy. This is because the complex lighting modeling in CR images provides depth information about adjacent structures and clarifies the relationship between anatomical structures, allowing for accurate spatial relationship assessment. These advantages make VR imaging more useful, especially when slices are thin and overlap images are not available. It can also be seen that VR images show wavy artifacts throughout the image. When the slice thickness is thick, the voxel values are averaged to reduce the spatial resolution, and it is much more difficult to evaluate the spatial relationship when post-processing the image in VR.

The BRISQUE is a factor that can properly evaluate the overall image quality of a CT image. When evaluating the image quality of medical images, including CT, the relationship between noise level and edge preservation ability must be carefully observed. Although the BRISQUE is a factor that can indirectly evaluate the edge area of an image, it has limitations in expressing accurate values. Tatsugami et al. showed the degree of preservation of the edge area by measuring the edge rise distance (ERD) as a distance value between 10% and 90% of the maximum Hounsfield unit value obtained from a certain ROI area in the CT image [33]. In the future, we expect that more accurate results will be presented if CT images using VR and CR technology are evaluated in a complex manner using ERD along with COV, which is a factor that can evaluate the noise level in this study. In addition, the representative no-reference-based image quality evaluation factor excluding BRISQUE is NIQE [34]. NIQE was modeled based on MSCN pre-processing, similar to the BRISQUE evaluation model used in this study. However, NIQE, unlike BRISQUE, is classified as an opinion-unaware method, a model that does not require a training process to match the image quality evaluation metric to the label. Thus, we plan to perform additional analysis on no-reference-based CT image quality evaluation factors by deriving NIQE results in the future.

This study has several limitations. First, the number of CT image samples used in the overall study is very small. In order to statistically analyze the advantages of CR technology based on the results of this study conducted under the pilot study concept, more sample data must be obtained. In addition, only anterior and posterior images were compared for all anatomical regions in this study. When rendering a CT image, data from various projection views can be utilized. We plan to obtain a lot of clinical information by additionally analyzing CT images from the lateral and oblique areas, along with anterior and posterior images.

## 4. Conclusions

This study evaluated the clinical usefulness of CR images through a no-reference method and noise level evaluations using bone CT. CR technology was able to derive excellent BRISQUE and COV values when reconstructing 3D CT images compared to existing VR technology. These results show that CR technology can improve the understanding of the spatial relationships of images owing to the reflection and shading caused by light characteristics.

## Figures and Tables

**Table 1:** Scan parameters of facial bone, shoulder, lumbar spine, and wrist CT images.

	Facial Bone	Shoulder	Lumbar Spine	Wrist
Tube voltage (kV)	100	110	120	120
Tube current (mAs)	120	117	224	99
Collimation width (mm)	0.6	0.6	0.6	0.625
Slice thickness (mm)	1	1	1	0.625
Interval (mm)	0.7	0.7	0.7	0.625
Pitch	0.8	0.8	0.8	0.9844

## Data Availability

The data that support the findings of this study are available from the corresponding author upon reasonable request.
